# Cognitive performance in healthy older adults relates to spontaneous switching between states of functional connectivity during rest

**DOI:** 10.1038/s41598-017-05425-7

**Published:** 2017-07-11

**Authors:** Joana Cabral, Diego Vidaurre, Paulo Marques, Ricardo Magalhães, Pedro Silva Moreira, José Miguel Soares, Gustavo Deco, Nuno Sousa, Morten L. Kringelbach

**Affiliations:** 1 Department of Psychiatry, University of Oxford, OX37JX Oxford, UK; 2 Center for Music in the Brain, Aarhus University, 8000 Aarhus, Denmark; 3 Oxford Centre for Human Brain Activity, University of Oxford, OX37JX Oxford, UK; 4 Life and Health Sciences Research Institute (ICVS), School of Medicine, University of Minho, 4710-057 Braga, Portugal; 5 ICVS/3B’s - PT Government Associate Laboratory, 4710-057 Braga, Portugal; 6 Clinical Academic Center (2CA-Braga), 4710-057 Braga, Portugal; 7 Center for Brain and Cognition, Computational Neuroscience Group, Universitat Pompeu Fabra, 08018 Barcelona, Spain; 8 Institució Catalana de Recerca i Estudis Avançats (ICREA), 08010 Barcelona, Spain; 9 Department of Neuropsychology, Max Planck Institute for Human Cognitive and Brain Sciences, 04103 Leipzig, Germany; 10 School of Psychological Sciences, Monash University, Clayton VIC 3800, Melbourne, Australia; 11 Institut d’études avancées de Paris, 75004 Paris, France

## Abstract

Growing evidence has shown that brain activity at rest slowly wanders through a repertoire of different states, where whole-brain functional connectivity (FC) temporarily settles into distinct FC patterns. Nevertheless, the functional role of resting-state activity remains unclear. Here, we investigate how the switching behavior of resting-state FC relates with cognitive performance in healthy older adults. We analyse resting-state fMRI data from 98 healthy adults previously categorized as being among the best or among the worst performers in a cohort study of >1000 subjects aged 50+ who underwent neuropsychological assessment. We use a novel approach focusing on the dominant FC pattern captured by the leading eigenvector of dynamic FC matrices. Recurrent FC patterns – or states – are detected and characterized in terms of lifetime, probability of occurrence and switching profiles. We find that poorer cognitive performance is associated with weaker FC temporal similarity together with altered switching between FC states. These results provide new evidence linking the switching dynamics of FC during rest with cognitive performance in later life, reinforcing the functional role of resting-state activity for effective cognitive processing.

## Introduction

Cognition involves the ability of recruiting specific functional networks, during which segregated brain areas form temporary coalitions to integrate and process information^1, 2^. Even at rest, when no task is performed, the brain displays the spontaneous waxing and waning of meaningful functional networks on a slow time scale (<0.1 Hz)^3–5^. This so-called *resting-state* activity has been proposed to reflect the spontaneous activation and deactivation of different network configurations supported by the structural *Connectome* ^6–9^, resulting in a constant reconfiguration of functional connectivity (FC) patterns over time. Importantly, this cannot be captured by traditional static FC analysis, where BOLD signal correlation is computed over the entire recording session^10, 11^.

Adding the temporal dimension to standard FC analysis paves new ways to characterize the switching behavior of resting-state activity^6, 9, 12–15^. However, the best methodology to assess it is still under debate. The most commonly used strategy has been to calculate successive *FC(t)* matrices using a sliding-window. Recurrent FC configurations are then captured by applying unsupervised clustering to all the *FC(t)s* obtained over time^6, 12^. However, the sliding-window approach has limitations associated to the window size, which affects the temporal resolution and statistical validation^13, 15–19^. Recently, new methods have been proposed to calculate the *FC(t)* at a quasi-instantaneous level, namely *Phase Coherence Connectivity* ^16, 20–22^ or *Multiplication of Temporal Derivatives* ^17^, which allow for a higher temporal resolution with the caveat of being more susceptible to high-frequency noise fluctuations^17^. To overcome this issue, we hereby propose to focus solely on the dominant FC pattern captured by the leading eigenvector of BOLD phase coherence matrices.

Focusing on the leading eigenvector of time-resolved FC matrices, we analyze the resting-state brain activity of healthy older adults free of cognitive impairment previously categorized as being among the best or among the worst performers in an extensive battery of neuropsychological tests^18, 19^. Indeed, different features of resting-state FC have been linked to cognitive fitness or intellectual performance^23–25^. In particular, in the context of healthy older adults, weaker correlations and decreased specificity of functional networks have been associated with decreased cognitive performance^26–32^. Nevertheless, all these studies refer to static aspects of resting-state FC and the relationship with spontaneous switching between brain states remains mostly unexplored.

## Methods

### Participants

Participants were selected from a cohort study where a sample of n = 1051 subjects aged 50+ years (representative of the Portuguese elder population in terms of gender and education) was previously characterized with an extensive battery of neuropsychological tests^18, 19, 33^. This neuropsychological assessment was carried out by certified psychologists and included the following tests: Digit-span Forward (DF) and Backward (DB) test, Stroop Words (SW), Stroop Colors (SC), Stroop Words/Colors (SWC), Controlled Oral Word Association Test (COWAT-FAS; admissible words), Selective Reminding Test (SRT), Digit Symbol Substitution Test (DSST), Mini-Mental State examination (MMSE), and Geriatric Depression Scale (GDS, long-version)^19^. Using Principal Component Analysis (PCA) applied to all neuropsychological data, two main dimensions of cognitive performance were identified, one being related to memory (MEM) and the other to general executive functioning (GENEXEC), while MMSE and GDS scores did not form any grouping with other neuropsychological variables^18^. Based on the scores in these dimensions and on the MMSE and GDS scores, cluster analysis revealed four clusters, sorted according to cognitive performance as C1 > C2 > C3 > C4, with C1 and C4 corresponding to the best and worst cognitive profiles^18^. Of note, GDS total score was a key variable for the separation between very good (C1) and good (C2), as well as poor (C3) and very poor (C4) performers (see ref. 18 for full details).

Two groups of participants were formed by randomly selecting n = 60 subjects from each of the abovementioned C1 and C4 profiles resulting in two groups with opposite cognitive profiles: 60 subjects with overall *good* cognitive performance and 60 subjects with overall *poor* cognitive performance. Differences between groups with respect to socio-demographic and cognitive measures are summarized in Table S1 in Supplemental Material. This subsample was then recruited to participate in the MRI scanning session for this study. Primary exclusion criteria included inability to understand the informed consent, participant’s voluntary withdrawal from the study, incapacity and/or inability to attend the MRI session, dementia and/or diagnosed neuropsychiatric and/or neurodegenerative disorder (medical records). From the subsample of 120 subjects, nine refused to undergo MRI screening, four had previously undiagnosed brain lesions/pathologies, and nine subjects were excluded due to excessive motion. A total of 98 subjects comprised the final sample, 55 good cognitive performers and 43 poor cognitive performers. This study was performed in accordance with the Declaration of Helsinki (59th amendment) and approved by national and local ethics review boards (Comissão Nacional de Protecção de Dados, Hospital de Braga, Centro Hospitalar do Alto Ave and Unidade Local de Saúde do Alto Minho). All volunteers signed informed consent and all medical and research professionals who had access to participants’ identity signed a Statement of Responsibility and Confidentiality.

### Functional MRI data

Prior to the acquisition, participants were instructed to remain still with eyes closed, not to fall asleep and not to think of anything in particular. The fMRI acquisition was performed using a clinical approved 1.5T Siemens Magnetom Avanto (Siemens Medical Solutions, Erlangen, Germany) MRI scanner with a 12-channel receive-only head coil at Hospital de Braga (Portugal). A BOLD sensitive echo-planar imaging (EPI) sequence was used with the following parameterization: 30 axial slices, TR/TE = 2000/30 ms, FA = 90°, slice thickness = 3.5 mm, slice gap = 0.48 mm, voxel size = 3.5 × 3.5 mm^2^, FoV = 1344 mm and 180 volumes.

Resting-state fMRI data preprocessing was performed with FMRIB Software Library (FSL v5.07; http://fsl.fmrib.ox.ac.uk/fsl/) tools^34–36^. The preprocessing steps included: 1) removal of the first five volumes of the acquisition in order to allow for signal stabilization; 2) slice timing correction; 3) motion correction with rigid body alignment of every volume to the mean image of the acquisition using MCFLIRT^37^; 4) skull stripping with the Brain Extraction Tool^38^ (BET); 5) non-linear normalization through the consecutive rigid-body registration of the functional acquisition to the structural acquisition using FLIRT, non-linear registration from structural native space to MNI standard space and resampling to 2 mm isotropic voxel size using FNIRT^39^; 6) linear regression of motion parameters, mean CSF and WM signals; 7) band-pass temporal filtering (0.01–0.08 Hz) of the residuals of the regression.

Mean BOLD time-series were then estimated on 90 brain areas of the Anatomical Automatic Labeling (AAL) atlas^40^ by averaging the BOLD signal over all voxels belonging to each brain area.

### Structural MRI data

A T1-weighted magnetization prepared rapid gradient echo (MPRAGE) acquisition was also performed with the following parameterization: 176 sagittal slices, TR/TE = 2730/3.48 ms, FA = 7°, slice thickness = 1 mm, slice gap = 0 mm, voxel size = 1 × 1 mm^2^, FoV = 256 mm. This acquisition was used as auxiliary for the spatial normalization of the functional scans. Structural scans underwent skull stripping using BET and non-linear registration from structural native space to MNI standard space followed by resampling to 2 mm isotropic voxel size.

### Diffusion MRI data

The Diffusion-Weighted Imaging (DWI) scan was performed in the same imaging session as the rs-fMRI scan and the structural scan. A spin-echo echo-planar imaging (SE-EPI) sequence with the following parameters was used: 61 axial slices, TR/TE = 8800/99 ms, slice thickness = 2 mm with no gap, voxel size = 2 × 2 mm^2^, FoV = 240 × 240 mm, 30 non-collinear gradient directions with b = 1000 s/mm^2^, one b = 0 s/mm^2^ acquisition and 2 repetitions. The DWI acquisitions were corrected for motion and eddy-current induced distortions using FSL’s tool *eddy_correct* and the B-matrix was rotated accordingly using the *fdt_rotate_bvecs* tool from the same distribution^41^. Additionally, skull striping was performed with BET in order to remove non-brain structures^38^.

### Structural Connectomes

Individual structural networks were built from DWI using probabilistic tractography combining BEDPOSTX and PROBTRACKX with the Fdt toolbox in FSL (www.fmrib.ox.ac.uk/fsl)^42, 43^, following the methods described in Cabral, *et al*.^44^. The AAL atlas was used for brain parcellation into N = 90 non-cerebellar brain areas^40^.

The individual Structural Connectomes (﻿SC) are *NxN* matrices where each entry *SC(n, p)* = *SC(p, n)* is weighted in proportion to the number of fiber tracts sampled in area *n* that reach area *p*, with *n*, *p* = 1, …, *N*. No threshold was applied. The individual SC were averaged across subjects resulting in a single SC representative of the whole population.

### Static Functional Connectivity

**Figure 1:**
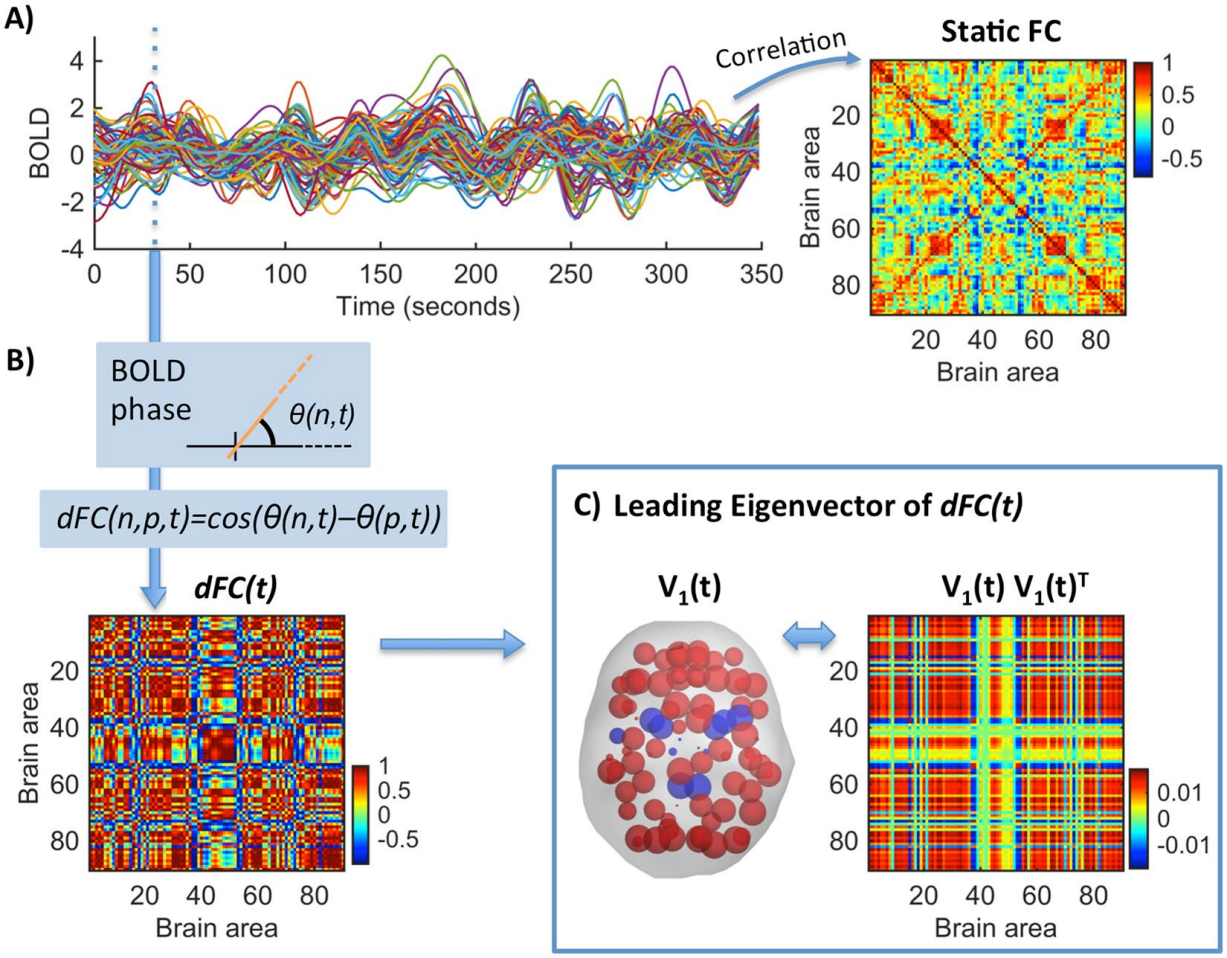
Time-resolved *dFC* and its Leading Eigenvector *V*_1_. (**A**) Resting-state BOLD signals from one subject at N = 90 brain areas. The traditional (static) FC matrix represents the correlation of BOLD signals over the whole recording time. (**B**) The *dFC* is obtained using BOLD Phase Coherence Connectivity^20^, such that each entry *dFC(n*, *p*, *t)* corresponds to the phase coherence between the BOLD signals in areas *n* and *p* at time t. At each time *t*, the *dFC(t)* is a symmetric *NxN* matrix. (**C**) The leading eigenvector, *V*_*1*_ *(t)*, captures the dominant connectivity pattern of *dFC(t)* at time *t*. We illustrate this pattern in two ways: (Left) We use *V*_*1*_ *(t)* to scale the size of spheres placed at the center of gravity of each brain area, coloring alike elements with the same sign. (Right) We plot the eigenvector’s outer product *V*_*1*_ *V*_*1*_^*T*^ (see *Methods* - *FC Leading Eigenvector*).

The FC between two brain regions is measured as the Pearson (zero-lag) correlation between their BOLD signals over the recording time. The FC between *N* = 90 brain areas is represented by a *NxN* FC matrix. Since this matrix collapses the temporal dimension into a single average, we refer to it as *Static FC* (see Fig. 1A).

### Dynamic Functional Connectivity

We use BOLD Phase Coherence Connectivity^16, 20–22^, to obtain a time-resolved *dynamic FC* matrix, or *dFC*, with size *NxNxT*, where *N* = 90 is the number of brain areas and *T* = *175* is the total number of recording frames. To compute the phase coherence, we first estimate the phase of the BOLD signals in all areas *n*, *θ(n*, *t)*, using the Hilbert transform (see Fig. 1B and Supplementary Figure S1). Given the phases of the BOLD signals, the phase coherence between brain areas *n* and *p* at time *t, dFC(n*, *p*, *t)*, is obtained using Equation (1):

1 $$dFC(n,p,t)=\,\cos (\theta (n,t)-\theta (p,t))$$

where *cos()* is the cosine function. Because *cos(0)* = *1*, if two areas *n* and *p* have temporarily aligned BOLD signals (i.e. they have similar phases), then *dFC(n, p, t)* will be close to 1. Instead, in periods where the BOLD signals are orthogonal (for instance, one increasing at 45° and the other decreasing at 45°) *dFC(n, p, t)* will be close to 0. Since the phase coherence is undirected, the *NxN dFC(t)* matrix is symmetric across the diagonal and hence all meaningful values can be captured from the upper (or lower) triangular parts of the matrix.

### FC Leading Eigenvector

To compare the *dFC* patterns over time, the most common approach is to compare the *NxN dFC(t)* matrices obtained at each time point. Since matrices are symmetric, comparison is typically performed between the upper triangular elements of the matrices^6, 14, 15, 22^. Here, we propose an alternative method where we consider only the leading eigenvector *V*_*1*_ *(t)* of each *dFC(t)* (see Fig. 1C for an illustration). The leading eigenvector *V*_*1*_ *(t)* (of dimension *Nx1)* captures the dominant connectivity pattern of *dFC(t)* at time *t*, which can be reconstructed using the (NxN) outer product *V*_*1*_ *V*_*1*_^*T*^. Compared to considering all (upper triangular) elements of *dFC* matrices, this approach strongly reduces the dimensionality from *N(N−1)/2* to *N* while still explaining most of its variance (see Supplemental Figure S2).

The eigenvectors of connectivity matrices are widely used to find community structures in networks^45, 46^, where network partition is performed by separating all eigenvector elements with positive sign from the elements with negative sign. Although community detection is beyond the scope of this work, it serves to illustrate the connectivity pattern captured by the leading eigenvector. For instance, in Fig. 1B each brain area *n* is assigned to one of two communities (blue or red) according to the corresponding sign in *V*_*1*_ *(n)* (note that *V* and −*V* represent the same eigenvector, so only the relative sign between regions is relevant). Moreover, the magnitude of eigenvector elements indicates the ‘strength’ with which brain areas belong to the communities in which they are placed^45^.

### Functional Connectivity Dynamics

**Figure 2:**
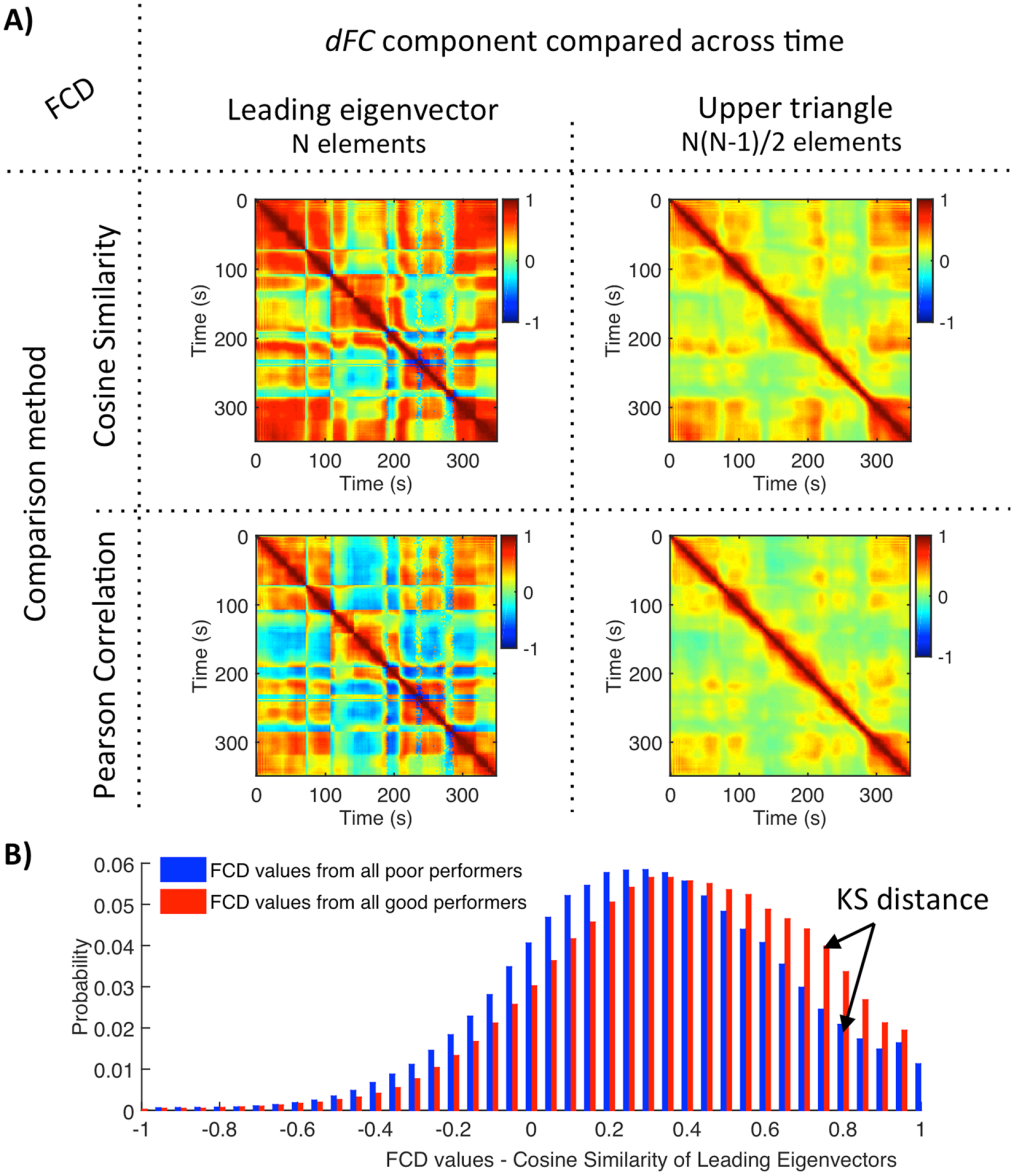
*FCD* methods and analysis. (**A**) In order to capture the time-dependencies of the *dFC*, each entry *FCD(t*_*x*_, *t*_*y*_ *)* contains a measure of resemblance between the *dFC* at times *t*_*x*_ and *t*_*y*_. This resemblance, is assessed by comparing different components of the *dFC(t*) (leading eigenvector, left column; upper triangular elements, right column) using either the cosine similarity (top line) or the Pearson correlation (bottom line) between components. (**B**) Probability densities of *FCD* values of all good and poor performers (*N*_*poor*_ = 43, *N*_*good*_ = 55) obtained using the cosine similarity of leading eigenvalues. Although all methods reveal the same temporal structure, the cosine similarity results in a better distinction between groups (i.e. larger Kolmogorov-Smirnov (KS) distance) and the leading eigenvectors capture better long-term recurrences of the same FC pattern.

To study the evolution of the *dFC* over time, we compute a time-versus-time matrix representing the functional connectivity dynamics (*FCD*), where each entry, *FCD(t*_*x*_ *, t*_*y*_ *)*, corresponds to a measure of resemblance between the *dFC* at times *t*_*x*_ and *t*_*y*_. As shown in Fig. 2, comparison between *dFC(t*_*x*_ *)* and *dFC(t*_*y*_ *)* is performed using either Pearson correlation or cosine similarity, and applied either to the *N(N−1)/2* upper triangular elements of *dFC(t)* or just to the *N* elements of its leading eigenvector *V*_*1*_ *(t)*. The measure of cosine similarity between the eigenvectors is given by Equation (2):

2 $$FCD({t}_{x},{t}_{y})=\frac{{V}_{1}({t}_{x})\cdot {V}_{1}({t}_{y})}{\Vert {V}_{1}({t}_{x})\Vert \Vert {V}_{1}({t}_{y})\Vert }$$

The cosine similarity corresponds to the inner product between two vectors divided by their norms, which results in a *bounded* inner product with values between −1 and 1.

### FC states

**Figure 3:**
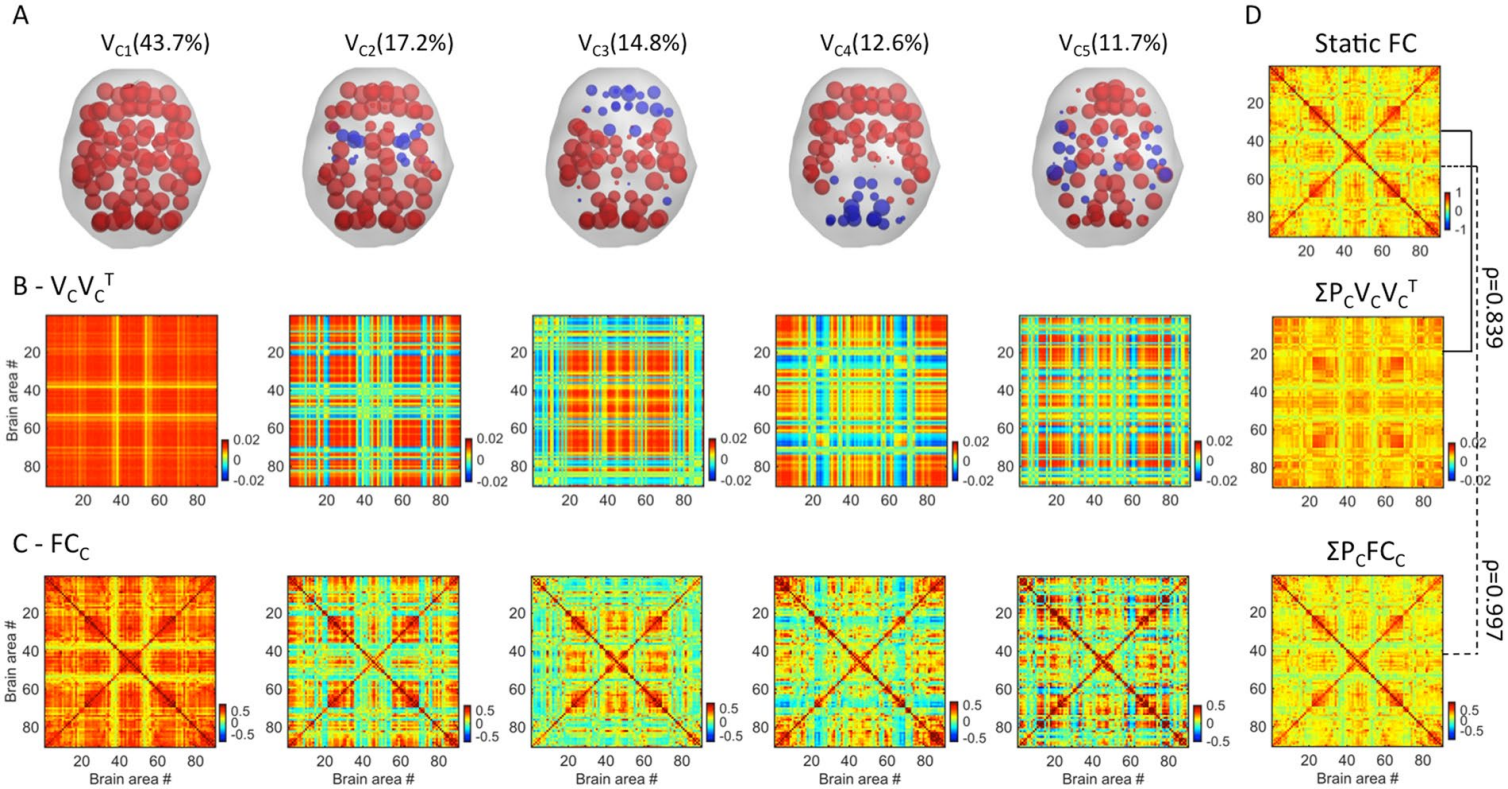
FC states and comparison with Static FC. Five recurrent FC patterns, or states, were obtained from clustering the leading eigenvectors of the *dFC*s of all participants. (**A**) Each of the five FC patterns is represented by a vector *V*_*C*_, where *V*_*C*_ *(n)* weighs the contribution of each brain area *n* to that pattern (displayed in cortical space). Elements with the same sign in *V*_*C*_ are colored alike to illustrate the network partition captured by *V*_*C*_ (see *Methods - FC Leading Eigenvector*). States are ranked according to their probability of occurrence (P_C_, in %). (**B**) *V*_*C*_ *V*_*C*_^*T*^ illustrates the *NxN* connectivity pattern corresponding to each state. (**C**) *dFC* averaged over the time points of each state. (**D**) The static FC averaged over all subjects (top) correlates strongly (*r* = 0.839) with the weighted sum of the five connectivity patterns captured by *V*_*C*_ *V*_*C*_^*T*^ (middle). The correlation increases up to *r* = 0.997 when considering the weighted sum of the *dFC* averaged over the time points of each state (bottom).

A discrete number of FC patterns is detected by applying clustering analysis on all the leading eigenvectors *V*_*1*_ *(t)* across time points and subjects (i.e. 175 × 98 = 17150 leading eigenvectors). We use *k*-means clustering with *k* (number of clusters) from 2 to 20, repeating each 20 times. As a result, we obtain *k* cluster centroids, each being a *Nx1* vector representing a recurrent FC pattern (see Fig. 3A). For each *k*, the clustering solution is evaluated using the Dunn’s score^47^. For comparison, we run the same analysis using the Hidden Markov model (HMM)^48^ instead of *k*-means clustering. Unlike *k*-means, the HMM accounts for the time-dependencies of the data.

### Between group comparisons

We use a permutation-based paired t-test to identify significant differences between groups. This non-parametric two-sample hypothesis test uses permutations of group labels to estimate the null distribution instead of relying on the test-type standard distributions. The null distribution is computed independently for each population. For each of 5000 permutations a t-test is applied to compare populations.

### Data and code availability statement

All data is available on request. The codes are publicly available at github.com/juanitacabral/LEiDA.

## Results

### FCD analysis

To better evaluate the temporal dependencies of the *dFC*, we start by comparing different approaches to obtain the *FCD* matrices (see Fig. 2A). Irrespective of the method, the *FCD* matrices reveal a characteristic checkered pattern indicative of spontaneous switching between different recurrent FC configurations. Large red squares in the diagonal of the *FCD* matrix represent stable *FC* configurations that tend to re-appear in non-contiguous time segments (i.e. in the same line/column but further from the diagonal) with sharp switches indicating a change of pattern.

When comparing only the leading eigenvectors of the *dFC* over time (left column in Fig. 2A), we find FC patterns to switch quite abruptly. Crucially, focusing on the leading eigenvectors allows us to detect the precise epochs when the variance of the *dFC* matrix becomes dominated by a different FC pattern, even when the *dFC* variation is smoother. Indeed, as shown in Fig. 2, similar *FCD* temporal profiles with smoother switches are found when looking at the whole upper triangular part of the *dFC* matrix (right column in Fig. 2A). Because it is less susceptible to noise, our approach also improves the sensitivity to pattern recurrences. For instance, in the example in Fig. 2A, the leading eigenvectors at t = 24 seconds and t = 344 seconds reach a similarity of 0.85 (more than 300 seconds later), whereas the whole *dFC(t)* matrices are less similar (0.65).

To investigate the differences between participants with the best and worst cognitive scores we compare the probability densities of all *FCD* values across groups (Fig. 2B). In order to do this, for each method, we evaluate how much the distribution of *FCD* values differs between good and poor performers using a 2-sample Kolmogorov-Smirnov (*KS)* test (see Fig. 2B). All methods reveal that good performers have significantly higher *FCD* values at rest (rejecting the null-hypothesis with *p* < 10^−15^), indicating that better cognitive performance in healthy older adults is associated to an overall stronger temporal similarity in the *dFC*, i.e. less temporal variability. Compared to Pearson correlation, the cosine similarity results in a better distinction between good and poor performers (*KS* distance using Cosine Similarity = 0.100 (leading eigenvector) and 0.105 (upper triangle); KS-distance using Pearson Correlation = 0.028 (leading eigenvector) and 0.044 (upper triangle)).

To understand whether stronger temporal similarity in the *dFC* of adults with the highest cognitive scores is due to more stable FC configurations, more frequent recurrences or even to higher prevalence of some FC patterns over the others, in the following we perform a deeper investigation into the switching behaviour between FC states.

### FC states

We identify five representative FC patterns –or FC states - in the resting-state activity of all 98 subjects (Fig. 3). More specifically, a *k*-means clustering algorithm was applied to the whole set of *dFC* leading eigenvectors and k = 5 returned as the best number of FC patterns representing the data (see Methods and Supplemental Figure S3). Each of the five cluster centroids (or states) is a vector *V*_*C*_, where *V*_*C*_ *V*_*C*_^*T*^ represents a *NxN* connectivity pattern and *V*_*C*_ *(n)* weighs the contribution of each brain area *n* to that pattern (see Fig. 3A,B and Figure S4 in *Supplemental Material*). FC states are ranked 1 to 5 according to their probability of occurrence, *P*_*C*_. We then compute the weighted sum of these *V*_*c*_ *V*_*c*_^*T*^ matrices according to *P*_*c*_, and find that it strongly correlates (ρ = 0.839) with the Static FC matrix averaged over all subjects (Fig. 3D), meaning that the Static FC matrix can be fairly represented as a linear combination of only 5 (eigen)vectors. In addition, we use the cluster time-courses to calculate the average *dFC* matrices over the time points represented by each cluster, providing more realistic FC patterns (Fig. 3C). Notably, the weighted sum of *dFC* matrices almost perfectly matches the static BOLD FC matrix averaged over all subjects (ρ = 0.997), demonstrating that *Phase Coherence Connectivity* efficiently captures the coupling between BOLD signals (Fig. 3D).

The most prevalent FC pattern (*V*_*C1*_), which occurs more than 40% of the time, corresponds to a state of global BOLD coherence (all *V*_*C1*_ elements have the same sign, so the outer product, *V*_*C1*_ *V*_*C1*_^*T*^, is non-negative). In other words, during the epochs *t* when the *dFC* is mainly shaped by this pattern, the BOLD signals of all brain areas exhibit a strong coherence.

In the remaining FC patterns, *V*_*C*_ has elements with different signs, indicating that FC can be partitioned into two communities (illustrated in red and blue), with positive FC values *within* the community elements and negative FC values *between* communities (see *Methods – FC Leading Eigenvector*). In more detail, during FC states #2–5, the BOLD phases of some subsets of brain areas misalign from the rest of the network and temporarily align together, dividing the brain into two separated functional networks. Notably, we find that these functional networks are symmetric across the midline and represent different brain subsystems (see Fig. 3A). In FC pattern #2, we find a community consisting only of subcortical areas including the Hippocampus, the Amygdala, the Insula, the Pallidum and the Putamen. In FC pattern #3, the Anterior Cingulate, the Medial Frontal, the Angular Gyri and the left Posterior Cingulate form a functional network independent from the rest of the brain. Note that this includes typical seeds from the Default Mode Network^49, 50^. FC pattern #4 is characterized by two functional networks, one comprising occipital areas together with the Posterior Cingulate and the Precuneus and the other comprising all the remaining areas. Finally, FC pattern #5 represents a decoupling of temporal and parietal areas from frontal and occipital cortices.

**Figure 4:**
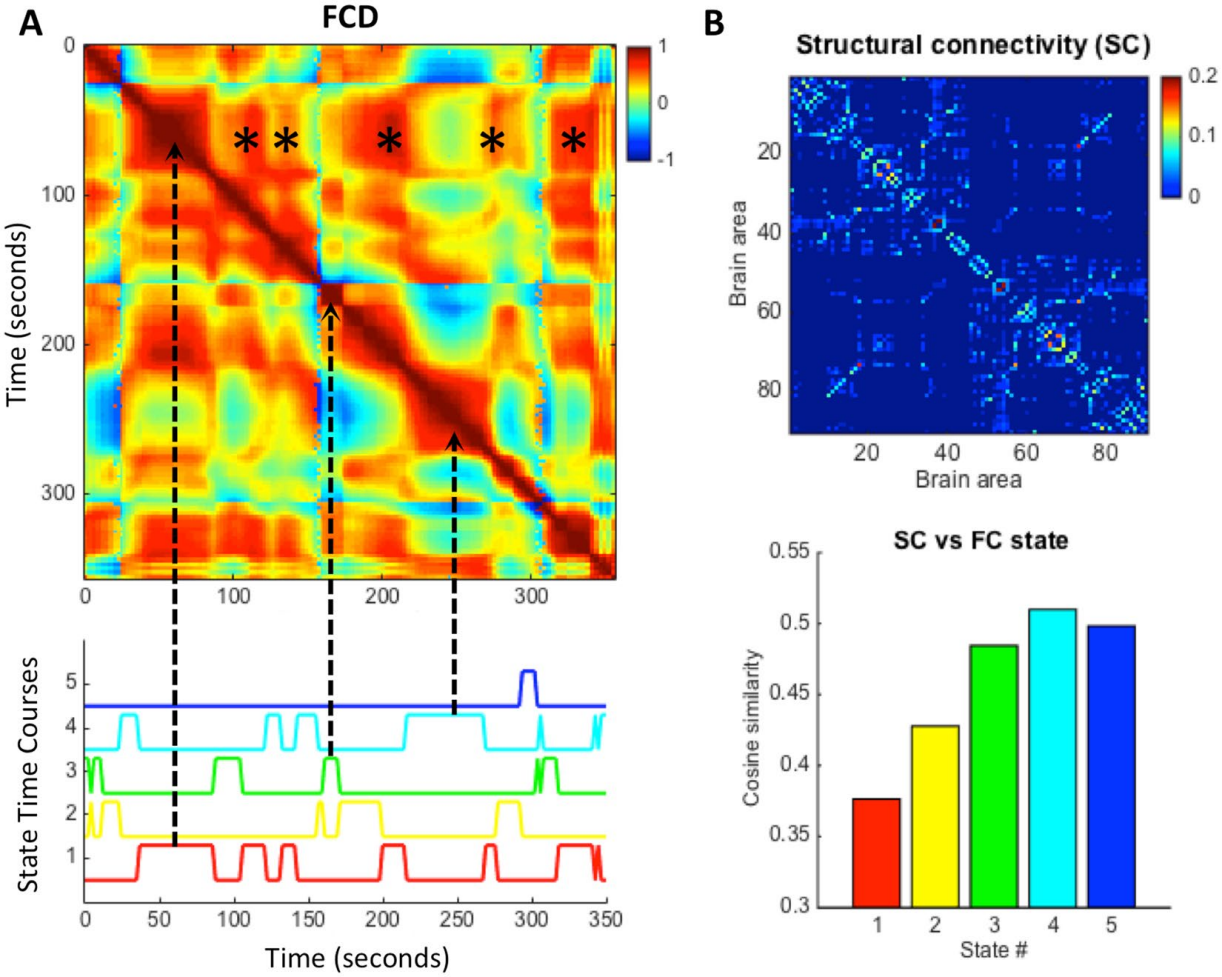
Correspondence of FC state time-courses with the *FCD* matrix and relationship with SC. (**A**) The time-versus-time *FCD* matrix (top) from one representative subject is compared with the corresponding FC state time-courses given by the *k*-means clustering algorithm (bottom). Note that each red square in the *FCD* matrix can be associated to the activation of a specific FC state (e.g. dashed arrows). Recurrent activations of FC state #1 (red time-course) are highlighted with (*) in the FCD matrix. (**B**) The structural connectivity matrix (SC) averaged over all subjects is compared with the mean FC_C_ matrices of each state by calculating the cosine similarity between values in the upper triangular parts of the matrices.

To compare the temporal behavior of FC states with the FCD matrices obtained before, we show in Fig. 4A an example of a *FCD* matrix (time-versus-time), displaying below the state time-courses over the same recording session. The state time-courses are obtained from the clustering algorithm and identify, for each epoch *t*, the cluster centroid (*V*_*C*_) that best approximates the leading eigenvector of the *dFC* at time *t*. One can easily observe that FC state transitions correspond nicely to switches in the *FCD* matrix (some of these correspondences are highlighted by arrows). In particular, with the use of the state time-courses, each red square in the *FCD* matrix can be associated to a specific FC pattern reoccurring over time (i.e. in the same line/column, as highlighted by asterisks for FC state 1). Moreover, the state time courses allow us to characterize the switching profile of FC states by computing measures such as the mean lifetime (i.e. number of consecutive epochs during which a given FC pattern dominates in variance), the switching frequency (i.e. number of transitions per second), the probability of occurrence of a given state and even the switching profiles (i.e. probabilities of switching from a given FC state to another), all of which cannot be assessed from the *FCD* matrix alone.

To investigate how the different FC patterns relate with the underlying anatomical network, we compared the mean *FC*_*C*_ of each state with the average SC of all subjects (Fig. 4B). Notably, although all the states share features of the structural network to some extent (similarity >0.37 for all states), the most prevalent FC patterns during rest are the ones that most differ from the SC matrix.

### Between-group differences in FC patterns

**Figure 5:**
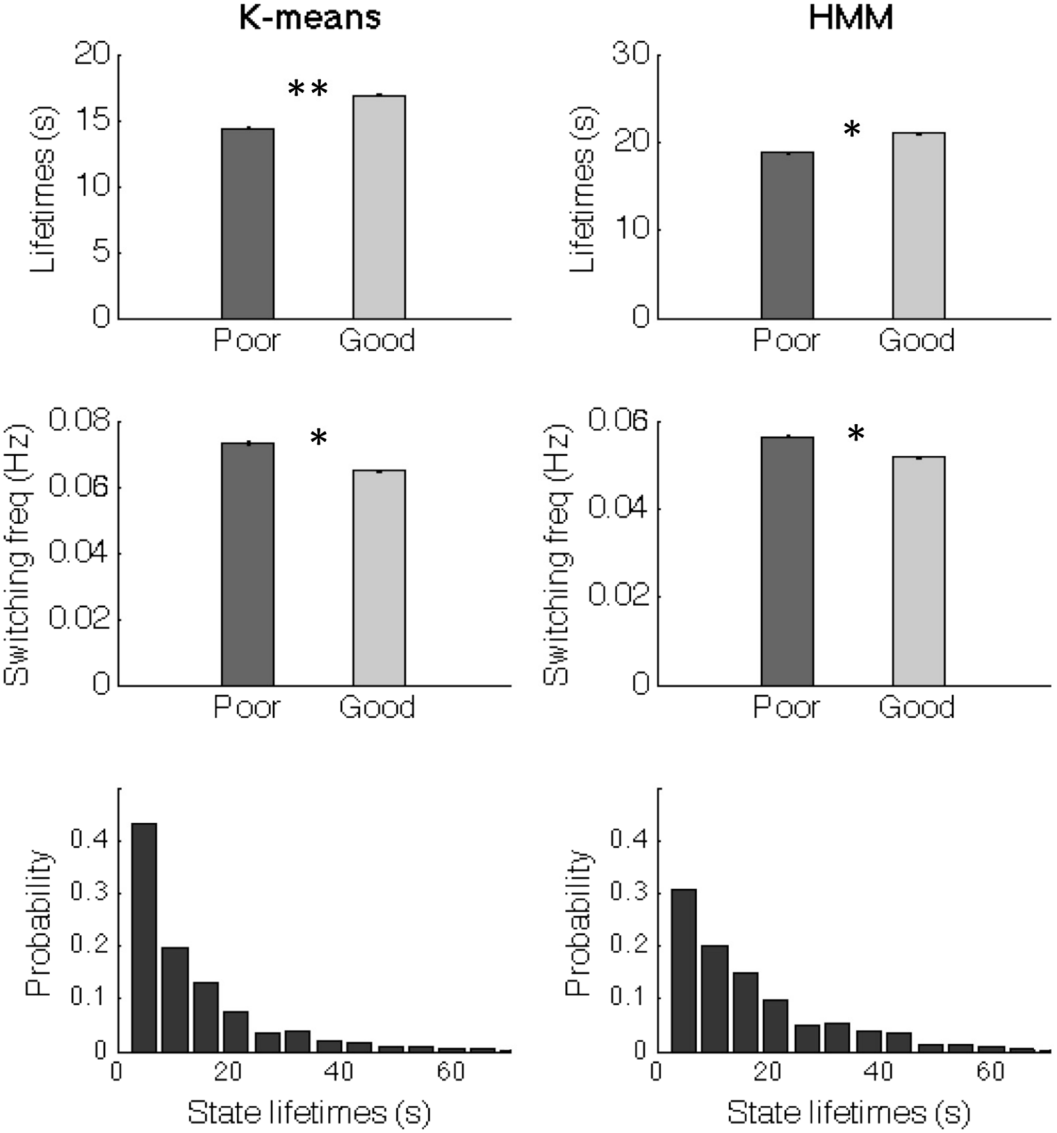
FC states last longer in participants with the best cognitive scores. (Top) Mean lifetime of FC states in seconds (s), counted as the mean time between transitions. The error bars indicate the standard error across subjects within each group of poor and good cognitive performers (* indicates *p* < 0.05). (Bottom) Probability density of FC state lifetimes. The same analyses were run using either the *k*-means clustering algorithm (left) or an HMM (right), with the same conclusions.

To investigate the relationship with cognitive performance, we compare the FC switching profiles between good and poor performers. In general, the resting-state FC in good performers is more stable in the sense that FC states last longer (mean ± standard error = 16.9 ± 0.73 seconds, p < 0.05) whereas FC states last shorter in poor performers (14.4 ± 0.59 seconds) (Fig. 5, left column). The associated switching frequency (1/Lifetime) falls in the range of the meaningful resting-state frequencies observed in BOLD fMRI data (<0.1 Hz)^51^, with good performers switching more slowly (mean switching frequency = 0.059 Hz) than poor performers (0.069 Hz).

To test the dependency of the results on the clustering algorithm, we run the same analyses substituting the *k*-means method by an HMM, which is explicitly designed to work on time series. Although the HMM states have slightly longer visits, all the conclusions remain valid (Fig. 5, right column).

**Figure 6:**
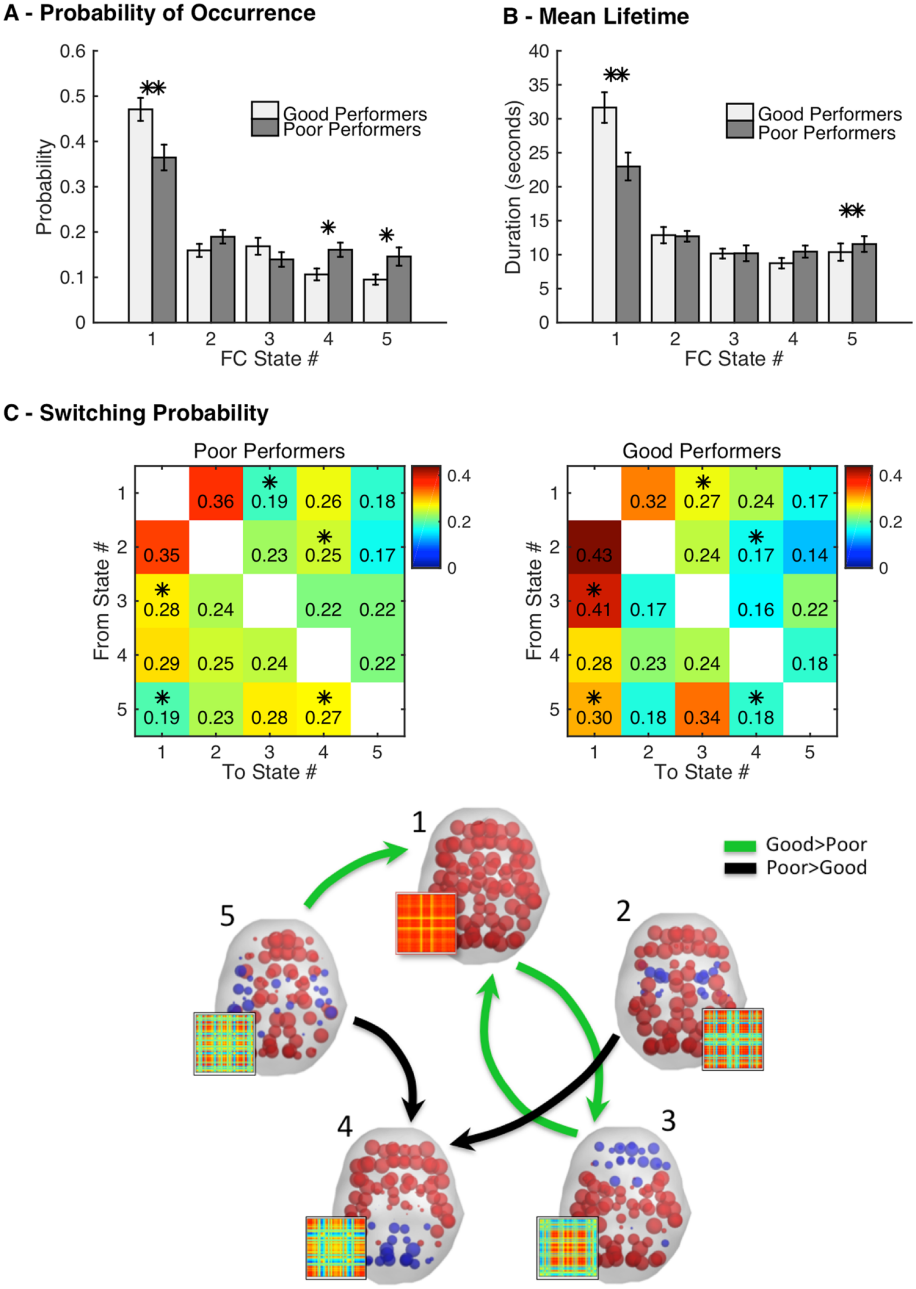
Switching between FC states relates with cognitive performance. (**A**) Fractional occupancy measured as the probability of occurrence of each state. The error-bars indicate the standard error. (**B**) Mean lifetime of each state. (**C**) Switching matrix indicating the probability of, being in a given FC state (lines), transitioning to any of the other states (columns). Significantly different transitions (p < 0.05) are illustrated in the plot below, with green arrows representing the transitions that occur with higher probability in good performers and in black the ones that occur with higher probability in poor performers. Each state is represented by the corresponding vector V_C_, displayed on cortical space (elements with the same sign in *V*_*c*_ are colored alike). The corresponding FC pattern is illustrated on the side (*V*_*c*_ *V*_*c*_^*T*^, see Methods). In A-B-C, values were estimated for each subject and then a permutation-based paired t-test was applied to test for the between-group significance. (*) and (**) indicate >95% and >99.5% confidence, respectively.

Focusing on the specific FC patterns (Fig. 6A,B), we find that good performers spend significantly more time in FC pattern #1 (47 ± 2.5% versus 36 ± 2.8% in poor performers, p < 0.005), with each occurrence lasting on average 31.7 ± 2.25 seconds, whereas poor performers only hold this state for around 22.97 ± 1.2 seconds (p < 0.05). Conversely, FC patterns #4 and #5 occur with more probability in poor performers (p < 0.05), with state #5 lasting longer in poor performers (p < 0.05).

Exploring the probability of switching from a given FC state to another, we find a number of differences between groups that pass a permutation-based paired t-test with *p* < 0.05 (Fig. 6C). First of all, being in state #1 the most probable transition is to state #2 in both groups. Once in state #2, the most probable switch is back to state #1 again, forming a closed loop between these two FC states. Switches from state #1 to state #3 are found to be more likely in good performers. Although the probability of occurrence of state #3 is similar in the two groups (Fig. 6A), once in state #3 the brain switches back to state #1 more frequently in good performers, forming another closed loop that appears less evident in poor performers. Looking at state #4, we find an increased switching probability from states #2 and #5 to state #4 in poor performers, which may explain why this state occurs more frequently in poor performers. However, once in state #4, the transition profile is very similar in the two groups. Finally, being in state #5, good performers transit most frequently to states #1 and #3 whereas poor performers switch predominantly to states #3 and #4.

## Discussion

In the present work, we find that the switching behavior of resting-state FC in healthy older adults relates to their performance in neuropsychological tests. These results are in line with the idea that brain activity during rest relates to higher order processes deeply involved in cognition and intellectual performance^23, 52–54^. In particular, we find increased temporal similarity in the resting-state FC of participants with the best cognitive scores. By investigating the switching profiles between FC states, we find that this is mainly due to the higher prevalence of a specific FC pattern over the others, which lasts shorter and occurs with less probability in participants with poor cognitive scores.

Our results show that the *Leading Eigenvector Dynamics Analysis* (*LEiDA*) introduced herein serves as a powerful tool to characterize the temporal evolution of the *dFC* with reduced dimensionality, which becomes useful in the analysis of multi-dimensional *dFC* data. Importantly, by focusing solely on its dominant connectivity pattern instead of the whole upper triangular part, *LEiDA* is more robust to high-frequency noise, overcoming a limitation affecting all quasi-instantaneous measures of FC^17^. Indeed, it allows detecting the precise epochs when the variance of the *dFC* becomes dominated by a different pattern, even if the *dFC* evolves more smoothly. Moreover, beyond significantly reducing the dimensionality of the data and allowing for improved temporal resolution, *LEiDA* offers the advantage that recurrences of the same pattern are more clearly detected, hence improving the signal-to-noise ratio in the FCD analysis.

Here, we find an optimal number of five dominant FC configurations where brain activity temporarily settles during rest. These results reinforce the idea that FC at rest is a multi-stable process^14^ where the connectivity patterns, rather than varying in a continuous sense, pass through multiple relatively stable FC states where the same pattern dominates in variance for more than 10 seconds. Note however that the number and shape of each FC pattern detected with LEiDA depends, among other factors, on the spatial and temporal scale over which it is defined^55^. Although the *dFC* matrices are calculated instantaneously, the BOLD signal is intrinsically slow (here in the narrow band 0.01–0.08 Hz) and we consider a coarse parcellation with only 90 non-cerebellar brain areas. Hence, the number of transiently recruited states reported herein refers to this specific context and population group.

The most prevalent FC state identified in this study is a state of global coherence of BOLD phases (FC state #1). This dominant mode of global BOLD co-variance is likely related to what is commonly described as the ‘global signal’ in resting-state fMRI studies, whose neurophysiological origin and role remains unclear^13, 56–59^. Here, we find a clear relationship between the prevalence of FC state #1 and cognitive performance, pointing to a potential role of global BOLD coherence in cognitive processing. The lower occurrence of this state in poor performers results in an overall weaker static BOLD FC, in line with previous findings where lower intelligence/poorer cognitive functioning was related to an overall decrease in BOLD functional connectivity in the elderly^28^.

In terms of FC-SC similarity, we find a nearly inverse relationship where the most prevalent FC patterns at rest are the least similar to the underlying SC. Notably, FC pattern #1 (more likely in good performers) is the most different from the SC, whereas patterns #4 and #5 (more likely in poor performers) are the most structurally shaped. Interestingly, these results suggest that cognitive functioning is related to a dissociation of FC from SC. Indeed, SC–FC similarity has been reported to decrease with conscious awareness in monkeys^60^ and, more recently, was found to increase from wakefulness to deep sleep in humans^61^.

Regarding the remaining FC states, it is tempting to compare them with pre-established resting-state networks (RSNs). However, RSNs are different from the FC states defined here, which refer to areas displaying correlated fluctuations over the whole recording time. That is, RSNs are temporal patterns that replicate across space while FC states are spatial patterns that replicate across time.

In the current approach, we consider only the leading eigenvector of the *dFC* and deliberately neglect the patterns captured by the remaining eigenvectors. This is justified by the fact that the leading eigenvector always represents more than 50% of the variance (i.e. its leading eigenvalue accounts for more than half of the total sum of eigenvalues). Yet, extending this method to consider a larger spectrum of eigenvectors deserves further attention.

It is worth noting that the clustering algorithm is just one among several different choices. A possible alternative to *k*-means is the Hidden Markov model (HMM), which allows for the specification of a tailored generative model and explicitly models the state transitions^48^. In this work, we have run the same analyses substituting the k-means method by an HMM, where each state is characterized by a Gaussian distribution with a state-specific mean and a global covariance matrix for all states. The conclusions presented above are reinforced by the fact that, except for having slightly longer state lifetimes, the results from the HMM are similar to what we obtained using k-means.

For a mechanistic interpretation of the results, it is useful to approach the problem from the perspective of dynamical systems’ theory^62–64^. Indeed, measures like the mean lifetime (or *dwell* time), the probability of occurrence (or *fractional occupancy*) and the switching paths (or *trajectories*) provide clues of the energy map of FC states where brain activity can temporally settle (or *FC attractor landscape)* ^64^. The detection of a number of re-occurring FC states is indicative of the number of attractors present in this landscape^6, 64^. Moreover, the fact that the system occasionally switches between states is indicative of being in a regime of *multi-stability* (i.e. the system never settles in a single, static FC state)^6^. Here, we find that lower cognitive performance is marked by decreased stability of FC state #1 together with increased stability of states #4 and #5, allowing the system to switch more often. Conversely, good performers show a higher probability of being in -or returning back to- FC state #1, which could be interpreted as a deeper and larger attraction basin associated to that state. Computational models of whole-brain network dynamics have shown that resting-state activity is optimally simulated when the system operates in a critical range of parameters where the system is continuously driven away from equilibrium and explores a repertoire of states^6, 8, 62, 64, 65^. In the context of dynamic FC, Hansen and colleagues^6^ enhanced the nonlinearities of the Dynamic Mean Field model^64^ to extend the repertoire of FC states in this critical regime and replicate the resting-state FC switching dynamics of healthy adults. For being in a critical regime, small changes in the system’s properties, such as alterations in local/global connectivity, excitation/inhibition, propagation speed, signal-to-noise ratio, etc., can directly affect the whole system’s dynamics^6, 7, 64, 66–70^.

Based on these previous theoretical and computational insights, we hypothesize that the differences in FC switching profiles found herein may be related to small changes in brain functioning gained throughout life due to factors such as education, socioeconomic status or engagement in cognitive demanding activities, or even factors with possibly a more acute effect such as mood. Indeed, clinical, physical, mood and lifestyle variables are known to influence age-related cognitive performance on various levels^18, 19^. Evaluations on the whole cohort of n = 1051 participants (from which the subsample of n = 98 participants considered here was extracted), found that education, age and depressed mood were the most significant variables in directly explaining the obtained cognitive score^18, 19^. In this study’s subsample, older adults categorized as good performers had more years of scholar education (7 ± 4 versus 4 ± 2 years, p < 10^−4^) and were less depressed (GDS = −0.371 ± 0.873 versus 0.471 ± 1.09, p < 10^−5^) than the group with poor cognitive performance (see Table S1 in the Supplementary Material). These findings go in line with current theories of cognitive reserve suggesting that education, occupation and social or mentally stimulating activities offer a protective effect against dementia^71, 72^. Previous analysis on the same dataset have shown that cognitive reserve is associated with a greater global efficiency of the functional connectome, which is subserved by enhanced connectivity between several brain regions^73^. Here, we show that cognitive performance of healthy older adults also significantly relates to their FC switching profiles during rest. On these grounds, FC switching measures may serve as novel indicators of cognitive reserve, allowing for earlier detection and better characterization of age-related cognitive changes from neuroimaging data.

## Electronic supplementary material

Supplementary Information
