# Supplementary material for: Cognitive performance in healthy older adults relates to spontaneous switching between states of functional connectivity during rest
Source: Sci Rep. 2017 Jul 11;7:5135. doi: 10.1038/s41598-017-05425-7 (PMC5506029; doi:10.1038/s41598-017-05425-7)
Supplement: Supplementary file 1 — Supplementary Information [file 41598_2017_5425_MOESM1_ESM.pdf]

Cognitive performance in healthy older adults relates to spontaneous switching between states of functional connectivity during rest

Joana Cabral, Diego Vidaurre, Paulo Marques, Ricardo Magalhães, Pedro Silva Moreira, José Miguel Soares, Gustavo Deco, Nuno Sousa, Morten L. Kringelbach

Supplementary Material

Table S1- Properties of good and poor performers. Cognitive data is presented in Z-scores.

	Good performers (N=55)	Poor performers (N=43)	Paired t-test (p-value)
Years old (mean ± std)	64 ± 9	66 ± 8	0.305
Sex (% males)	56.36%	32.73%	0.157
School years (mean ± std)	7 ± 4	4 ± 2	$<10^{-4}$ *
Cognitive tests:			
MEM (mean ± std)	1.31 ± 0.627	-0.801 ± 0.505	$<10^{-31}$ *
GENEXEC (mean ± std)	1.04 ± 0.890	-1.12 ± 0.600	$<10^{-23}$ *
MMSE (mean ± std)	0.727 ± 0.468	-0.438 ± 0.942	$<10^{-11}$ *
GDS (mean ± std)	-0.371 ± 0.873	0.471 ± 1.09	$<10^{-5}$ *

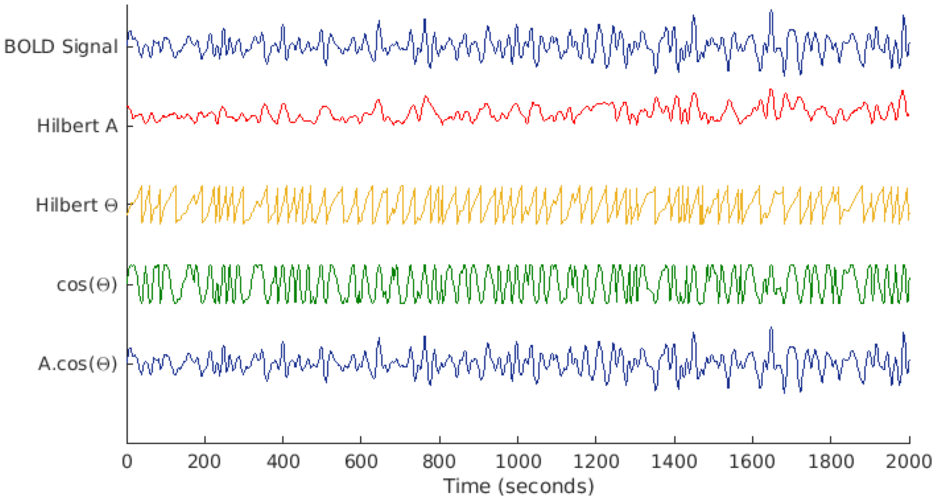

Figure S1 - The Hilbert transform expresses the BOLD signal (top line) in polar coordinates (bottom line). The temporal resolution of the Hilbert Phase and Amplitude is the same as the non-transformed BOLD signal and the correlation between the BOLD signal and *A.*cos(θ)* is 1.

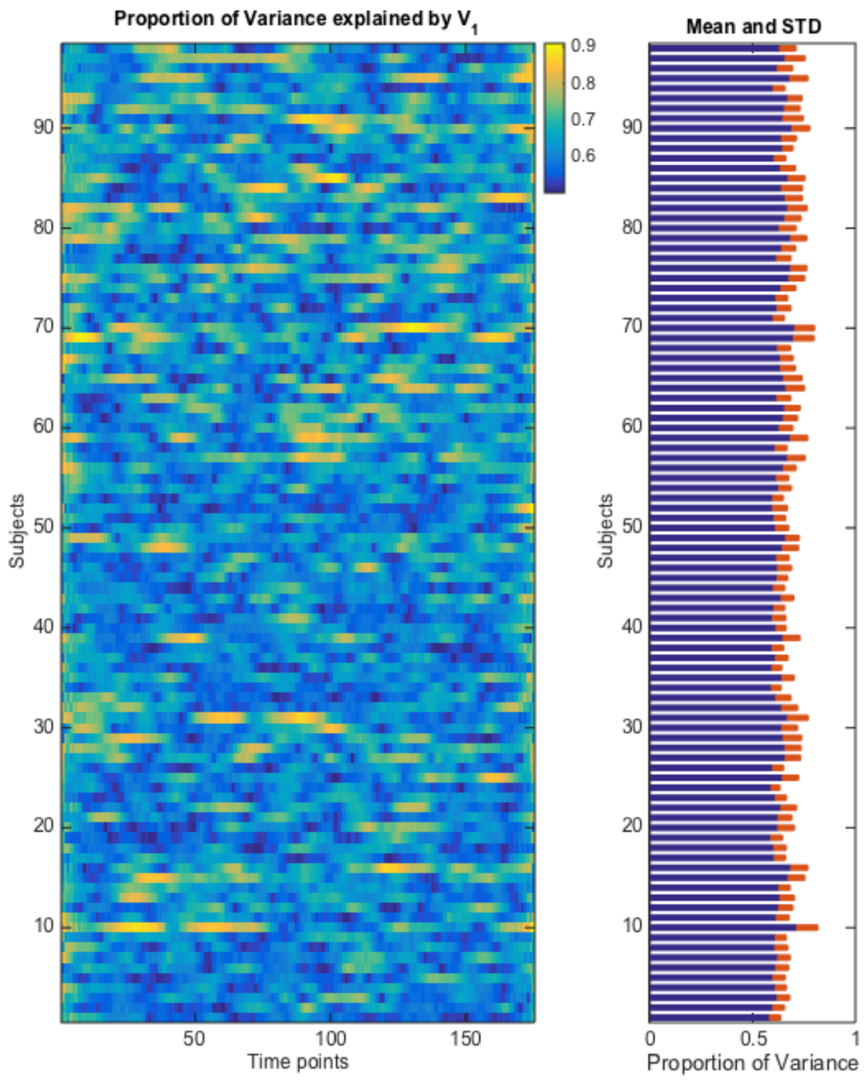

Figure S2 - Proportion of variance accounted for by the iFC leading eigenvector. The proportion of variance is obtained by dividing the leading eigenvalue by the sum of all eigenvalues. (Left) At all time points and for all subjects, the leading eigenvector always accounts for more than 50% of the variance, sporadically reaching 90%, with a mean of 63.4%. (Right) At the individual level, we find that the proportion of explained variance remains quite stable across subjects (STD, standard deviation in red). Subjects 1 to 43 are poor cognitive performers and 44 to 98 are good cognitive performers.

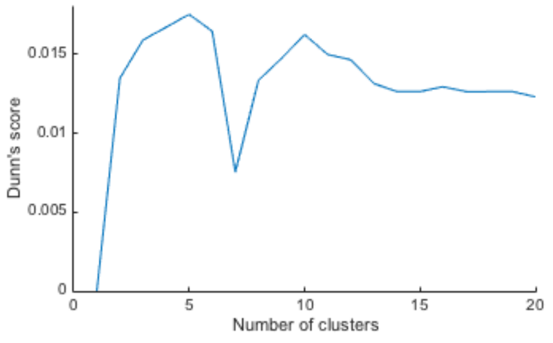

Figure S3 - Optimal Cluster solution. From the range of *k* assessed, *k*=5 maximized the Dunn's Index.

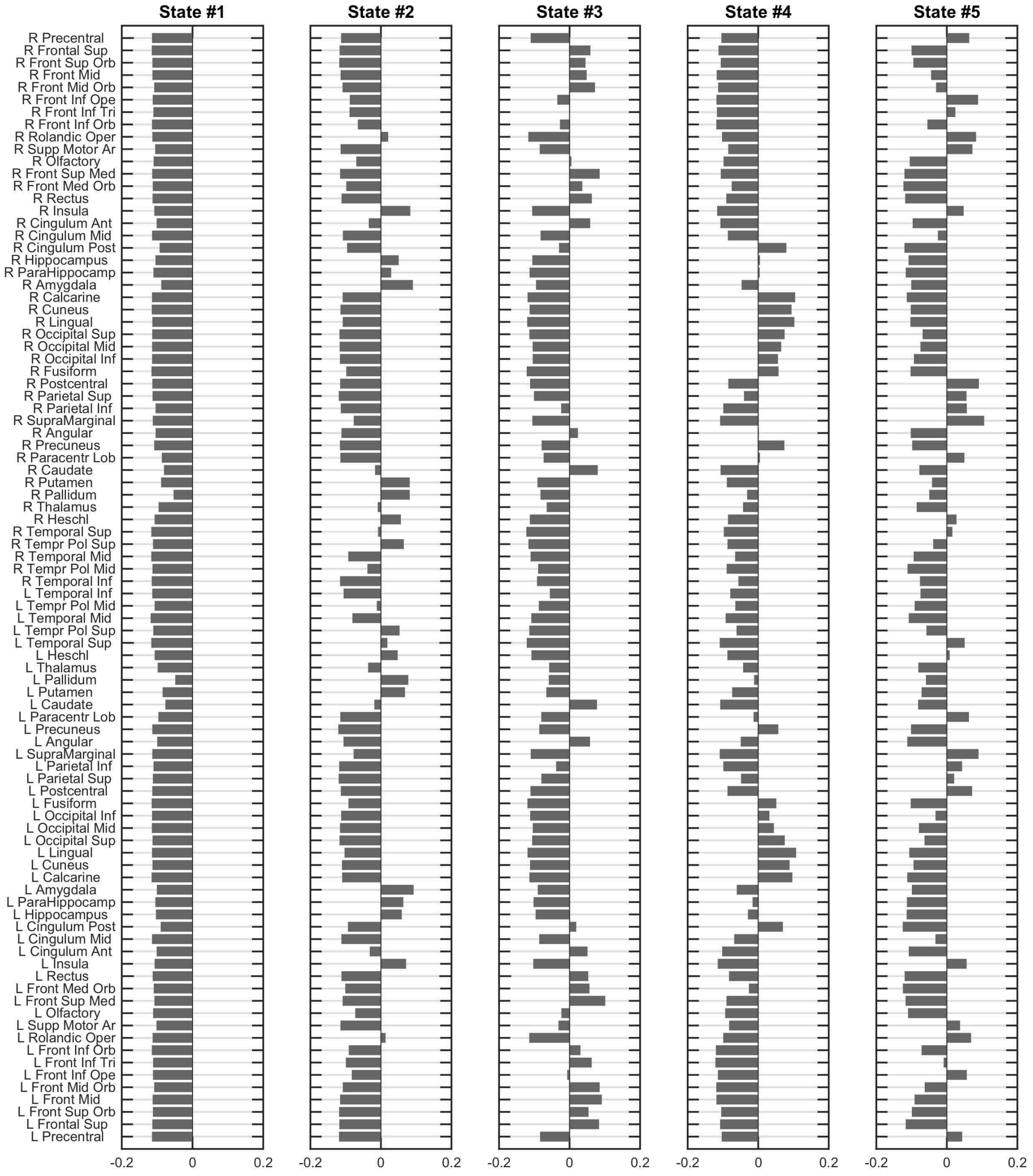

Figure S4 – Vectors selected by the clustering algorithm representing each FC state. The eigenvectors of connectivity matrices are commonly used to find community structures in networks following the method proposed by Newman [1], where nodes are partitioned into communities according to the element's sign in the eigenvector. The leading eigenvector captures the highest hierarchical partitioning of the network. When all elements have the same sign (state #1), areas behave coherently forming a global community in the highest hierarchical level. The magnitude of eigenvector elements (rows) indicates the 'strength' with which brain areas belong to the communities in which they are placed[1].

1 Newman, M. E. Finding community structure in networks using the eigenvectors of matrices. *Physical review. E, Statistical, nonlinear, and soft matter physics* **74**, 036104, doi:10.1103/PhysRevE.74.036104 (2006).
